# Roles of Arterial Baroreceptor Reflex During Bezold-Jarisch Reflex

**DOI:** 10.2174/157340309789317805

**Published:** 2009-11

**Authors:** Koji Kashihara

**Affiliations:** Furo-cho, Chikusa-ku, Nagoya, 464-8601, Japan

**Keywords:** Sympathetic nerve activity, arterial pressure, cardiopulmonary reflex, central pathway, acute myocardial ischemia.

## Abstract

Among the many cardiopulmonary reflexes, this review specifically examines the roles of the arterial baroreflex during the Bezold-Jarisch reflex (BJR). Activation of cardiopulmonary vagal afferent C-fibers induces hypotension, bradycardia, and apnea, which are known collectively as the BJR; myocardial ischemia and infarction might induce the BJR. Arterial baroreflex has been established as an important negative feedback system that stabilizes arterial blood pressure against exogenous pressure perturbations. Therefore, understanding the functions of the arterial baroreflex during the BJR is crucial for elucidating its pathophysiological implications. The main central pathways of the BJR and the baroreflex are outlined herein, particularly addressing the common pathway between the reflexes. Furthermore, the pathophysiological roles of the arterial baroreflex during the BJR are described along with a brief discussion of pathophysiological merits and shortcomings of the reflexes.

## INTRODUCTION

	Cardiorespiratory responses showing bradycardia, hypotension, and apnea through cardiopulmonary vagal afferent C fibers are known as the Bezold-Jarisch reflex (BJR). This reflex was observed initially subsequent to intravenous injection of veratrum alkaloids [1, 2]. Activation of the cardiopulmonary vagal afferent fibers during the BJR blunts efferent sympathetic nerve activity (SNA), thereby engendering hypotension and bradycardia [3-5]. The BJR might strongly affect developing pathophysiological respon-ses or circulatory regulation, potentially activating cardiac receptors. The BJR might be observed during acute myo-cardial ischemia or infarction [6-8]. However, the pathophysiological significance of this reflex remains controvertible [9, 10].

	Von Bezold and Hirt [1] initially proposed that intravenous injection of veratrum alkaloids induced hypotension and bradycardia in conjunction with apnea. Jarisch and Richter [2] reported that the powerful depressor action induced by intravenous veratridine was attributable to the cardiac branches of vagus nerves in cats. Subsequently, it has been established that this reflex originates mainly in the cardiopulmonary receptors on the unmyelinated and type C vagal afferent fibers [11-16]. Actually, the response of the BJR is abolished dramatically by bilateral vagotomy [1, 4, 17]. The vagus in the cardiopulmonary regions contains the population of chemo- and mechano-sensitive afferent C fibers, and arises from receptors located in the atria, ventricles, aorta, and lungs [18, 19]. Although the chemosensitive 5-HT_3_ receptors are generally known as the origin of the BJR, the mechanosensitive receptors might be also related to this reflex. Reportedly, the physiological responses to the stimulations of chemical and mechanical receptors in unmyelinated afferent C fibers are similar, respectively causing hypotension and bradycardia [20, 21]. The bradycardia and hypotension based on sympathetic vasomotor inhibition, and apnea mediated by vagal afferent C fibers from the cardiopulmonary area are today known by an extensive definition as the BJR [19, 22].

	Arterial baroreflex, on the other hand, is a crucial negative feedback system that quickly stabilizes arterial blood pressure (AP) against exogenous pressure perturbations [23, 24]. For instance, moving to a standing position causes rapid hypotension in the upper body and brain because of gravity, including the risk of losing consciousness. Arterial baroreflex originates in stretch receptors (baroreceptors) distributed in the walls of carotid sinuses and aortic arches, which are the origin of afferent fibers in the glossopharyngeal and vagal nerves. Increased AP induces expansion or contraction in the baroreceptors and facilitates the arterial baroreceptors’ afferent discharge to transmit signals to the central nervous system. The baroreceptor signals inhibit the vasoconstrictor center of the medulla and excite the vagal center, ultimately causing vasodilation through the peripheral circulatory system and blunted cardiac contractility.

	In contrast, decreased AP induces contraction in the baroreceptors and inhibits arterial baroreceptors’ afferent discharge. The feedback signals minimize AP disturbances within the normal level maintaining the systemic circulation, through the autonomic nervous system [23, 25]. Consequently, excitation of the afferent fiber originating from the baroreceptors by pressure change attenuates the heart rate (HR), cardiac output, and peripheral resistance, resulting in decreased AP. Especially in cardiovascular diseases, it is crucial to assess the ability of the arterial baroreflex to regulate AP against external pressure disturbances by pharmacological agents or positional changes.

	Excellent interpretations of various cardiopulmonary reflexes exist [e.g. 22,  24,  26-28]. This paper therefore specifically examines 1) the interactive roles of arterial baroreflex during the BJR, including 2) the common central pathways between the reflexes and 3) the pathophysiological roles of the BJR in acute myocardial ischemia.

## ARTERIAL BAROREFLEX DURING BJR

	In animal studies, the BJR has been chemically activated by pharmacological agents such as veratridine, nicotine, capsaicin, and a selective serotonergic 5-HT_3_ receptor agonist, phenyl biguanide (PBG) [29, 30]. As described herein, effects of the BJR induced by PBG were concentrated.

	Changes in SNA of kidney and AP during PBG infusion are mainly mediated by the activation of vagal afferent C fibers [4, 31]. Veelken *et al*. [32] reported that continuous PBG infusion attenuated SNA, AP, and HR during the first minute in conscious and anesthetized rats. Sympathoinhibition was maintained for 15 min, although AP and HR reverted to baseline values. In contrast, AP and HR with SNA decreased during 20-min PBG administration (i.v.) [4, 5], indicating the disappearance of the counteraction by the arterial baroreflex under the carotid-sinus open-loop condition.

### Static Property 

	Experimental studies have investigated the interaction between the BJR and the baroreflex. Veelken *et al*. [33] evaluated the baroreflex ability during the BJR by PBG infusion under the closed-loop AP response. The maximum gain of HR in the arterial baroreflex was impaired; the arterial baroreceptor control of SNA was not impaired during continuous PBG infusion, suggesting the remaining ability of the baroreflex. In contrast, the BJR attenuated the steady-state responses of SNA, AP, and HR to baroreceptor pressure input in anesthetized rabbits under the carotid-sinus open-loop condition [3, 5]. Chen [3] demonstrated that veratridine (i.v.) depressed the stepwise responses of HR to the carotid sinus pressure (CSP); the bradycardia was more prominent at lower CSP levels. In contrast, the response range of HR to CSP inputs might not be changed considerably, despite marked bradycardia [5].

	Intravenous PBG [5] as well as veratridine [3] blunted stepwise responses between the CSP and AP, attenuating the response range of AP. This result was attributable mainly to the decreased gain of static responses in the sympathetic outflow reflecting the action of the central region compared to those of the peripheral region. The peripheral gain might be much lower in acute myocardial ischemia or infarction because of pump failure, indicating a further decrease in AP and the reduced static gain.

### Dynamic Property 

	The BJR by intravenous PBG reduced the dynamic transfer gain to characterize the stability and quickness of AP regulation in the arterial baroreflex under a carotid-sinus open-loop condition in anesthetized rabbits [4, 34]. The result was due mainly to attenuating the dynamic gain in the central region [4]. Excess activation of the BJR during acute myocardial ischemia or infarction might exert inverse effects on AP regulation through attenuation of baroreflex dynamic gain as well as sympathetic suppression. Although PBG decreased the dynamic gain of the central region, the derivative characteristics were, interestingly, preserved around the operating point showing the normal AP. The dynamic gains at nonlinear CSP points such as hypertension and hypotension were attenuated during the BJR compared with that of the operating point [34].

## COMMON CENTRAL PATHWAY

	Because of recent studies using pharmacological agents to the related receptors, the central pathways during the BJR and the arterial baroreflex have been well identified. Overall, the results seem to show a similar pathway in the two reflexes.

### Main Central Pathways

#### Baroreflex 

	The sympathetic outflow in the baroreflex mainly passes through the brainstem such as the nucleus tractus solitarius (NTS), the caudal ventrolateral medulla (CVLM), and the rostral ventrolateral medulla (RVLM) [27, 35]. First, baroreceptor afferent fibers terminate within the NTS as the crucial site of the baroreflex [36]. The NTS projects directly to the CVLM through excitatory glutamatergic chemosensitive neurons [37, 38]. Through GABAergic neurons, the CVLM inhibits sympatho-excitatory neurons in the RVLM [39, 40]. Finally, the RVLM regulates the sympathetic vasomotor tone and the barosensitive neurons projecting to the spinal cord [27, 35]. Other important pathways such as medullary raphe nuclei and the lateral tegmental field are also related to this reflex [41].

#### BJR 

	Vagal afferent C fibers originating in the heart and lungs first terminate in the NTS [42]. Merahi *et al*. [43] demonstrated that most 5-HT_3_ receptors in the NTS are found on the vagal sensory afferent fibers using autoradiographic studies. Pires *et al*. [44] demonstrated that intracisternal or the NTS injection of the 5-HT_3_ receptor antagonist granisetron significantly attenuated the hypotension and bradycardia evoked by intravenous PBG, suggesting that the NTS is involved in the central pathway of the BJR.

	Intravenous PBG infusion to stimulate cardiac receptors facilitates firing of CVLM neurons, resulting in the SNA inhibition [45]*.* Schreihofer *et al*. [46] showed that an intravenous PBG infusion dramatically activated the GABAergic baro-activated CVLM neuron and caused the inhibited SNA. Verberne *et al*. [47] demonstrated that barosensitive neurons in the RVLM were inhibited by intravenous PBG in rats. It is conceivable that PBG attenuates the dynamic gain of the neural arc transfer function by affecting baroreflex signal transduction in such brainstem areas as the NTS, the CVLM, and the RVLM through activation of the vagal afferent C fibers.

	Verberne *et al*. [48] also showed the effects of the intravenous PBG on the neurons in rostrocaudal levels of the ventrolateral medulla (type I-VI). Cardiopulmonary receptor activation by PBG extremely suppressed the firing of barosensitive and bulbospinal neurons in the RVLM (type I), and produced excitation of the neuron in the CVLM site (type VI). These reports show direct evidence that the common sites are used for the baroreflex and the BJR. However, there might be specific differences among animals, as demonstrated in the baroreflex response in cats [41].

### Common Pathway

	As described above, the BJR and baroreflex signals appear to use common central pathways in brainstem regions such as the NTS, the CVLM, and the RVLM [47-49]. The glutamate receptor antagonist kynurenate into the NTS prevents the sympathoinhibition and hypotension by intravenous PBG infusion [47, 49, 50]. These results indicate that vagal afferent C fibers during the BJR use an excitatory amino acid neurotransmitter in the NTS, as shown in afferent baroreceptor fibers.

	In the CVLM, the excitatory amino acid antagonist attenuates the BJR by PBG infusion [47, 51]. Bicuculine, GABA receptor antagonist, into the RVLM attenuated the response of the BJR. However, kynurenic acid did not modulate it. In addition, the BJR induced by serotonin or PBG causes a sympathoinhibition with a decrease in the firing rate of barosensitive neurons in the RVLM [47, 52]. Consequently, the central pathway of the BJR clearly correlates with the NTS, the CVLM, and the RVLM with the excitatory and inhibitory amino acid neurotransmitters in the baroreflex.

## CLINICAL IMPLICATION

### BJR Induced by Myocardial Ischemia

	Actually, the BJR might strongly influence circulatory regulation during acute myocardial ischemia or infarction, when cardiac chemoreceptors are potentially activated [6, 7]. Elucidating the effects of the BJR on circulatory regulation is expected to contribute to the pathophysiological understanding in ischemic heart diseases [9, 10].

	The origin of the BJR by intravenous PBG might differ from that induced by myocardial ischemia. In cardiac diseases, serotonin levels might be increased in the coronary circulation [53]. On the other hand, an increase in the myocardial acetylcholine level induced by intravenous PBG is similar to that observed in the non-ischemic myocardium during coronary artery occlusion in anesthetized cats [54, 55]. These reports imply other reasons for the induction of the BJR in myocardial ischemia. Some reports describe oxygen-derived free radicals and prostaglandins generated during ischemia and reperfusion [56, 57]. Mechanoreceptors as well as chemoreceptors might also be activated during myocardial ischemia [6].

### Roles of BJR in Myocardial Ischemia

	Apparently, the BJR entails both the pathophysiological merits and shortcomings. Arrhythmias by bradycardia and hypotension, which show similar responses to that of BJR, are observed more remarkably in patients with inferior and posterior walls of the heart under myocardial ischemia and infarction [6, 9, 58]. Anatomically, the population of the cardioinhibitory C fiber afferent receptors in the myocardium correlates with the site of the inferoposterior walls of the left ventricle [59].

	The induction of the BJR might prevent overexertion of the cardiac muscle by bradycardia and hypotension [10]. The reduction of energy consumption might be beneficial for hampering ischemic insult and salvaging tissues in the ischemic border zone, indicating a heart-protective effect [9, 60, 61].

	The BJR attenuates the ability of AP regulation by the arterial baroreflex. Excess activation of the BJR might cause severe bradycardia and hypotension, placing the patient's life at risk because the magnitude of sympathetic inhibition and vagal activation during the BJR is not regulated in terms of AP regulation. Therefore, for patients with acute cardiac ischemia or infarction, clinicians should consider drug treatments causing a pressure change or change of a position with caution because of the blunted ability of the baroreflex during the BJR. Furthermore, the BJR might cause sudden cardiac death during ischemic injury [6] because severe hypotension eliminates the buffering function in the baroreflex regulation.

## CONCLUSION

	This report reviewed the baroreflex capability during the BJR that might be induced in myocardial ischemia or infarction. Through a vagal afferent pathway from the cardiac receptors, the BJR inhibited SNA, AP, and HR; the ability of the baroreflex was blunted especially in the central region such as the brainstem. Both the BJR and the baroreflex share similar central pathways-the NTS, the CVLM, and the RVLM-causing the reduction of dynamic and static gains.

	In acute myocardial ischemia or infarction, preventing severe hypotension during the BJR is necessary for stabilization of AP by the baroreflex system because, apparently, the natural property of life-the heart protection to prevent the local death of myocardium-is dominantly selected under such risky conditions, irrespective of blunting of the baroreflex ability and the possibility of sudden cardiac death. Further investigations related to central and peripheral regions are necessary to establish the pathophysiological mechanisms of the baroreflex and the BJR.
